# Evaluation of sheep exposure to poly- and perfluoroalkyl substances through hair sample analysis

**DOI:** 10.2478/jvetres-2025-0023

**Published:** 2025-04-04

**Authors:** Sławomir Gonkowski, Julia Martín, Nariste Kadyralieva, Irene Aparicio, Juan Luis Santos, Esteban Alonso

**Affiliations:** 1 Department of Clinical Physiology, Faculty of Veterinary Medicine, University of Warmia and Mazury, 10-957 Olsztyn, Poland; 2 Departamento de Química Analítica, Escuela Politécnica Superior, Universidad de Sevilla, E-41011 Seville, Spain; 3 Department of Histology and Embryology, Kyrgyz-Turkish Manas University, Veterinary Faculty, 720044 Bishkek, Kyrgyzstan

**Keywords:** endocrine disruptors, exposure, farm animals, hair, wool

## Abstract

**Introduction:**

Poly- and perfluoroalkyl substances (PFASs) are a numerous group of synthetic organic compounds used in various industries. They pollute the natural environment and negatively affect humans and animals. The aim of the present investigation was to assess the exposure of sheep bred in the Kyrgyz Republic to six selected PFASs.

**Material and Methods:**

Six selected PFASs were assessed in 50 sheep (39 females and 11 males) of Kyrgyz and Arashan breeds ranging in age from 1 to 8 years (mean 2.1 ± 1.1), recruited equally from the Alamedin and Sokuluk regions. The substances were five perfluoroalkyl carboxylic acids (perfluorobutanoic acid – PFBuA, perfluoropentanoic acid – PFPeA, perfluorohexanoic acid – PFHxA, perfluoroheptanoic acid – PFHpA and perfluorooctanoic acid – PFOA) and perfluorooctane sulphonic acid (PFOS), and were determined through the liquid chromatography–tandem mass spectrometry of hair samples.

**Results:**

All the listed PFASs were found in the studied hair samples. The highest concentrations were noted for PFPeA and PFBuA. The concentration of PFPeA ranged from 0.99 ng/g to 27.90 ng/g (mean 5.55 ± 4.54 ng/g) and that of PFBuA from 0.95ng/g to 14.18 ng/g (mean 2.24 ± 2.34 ng/g). The mean concentration levels of other PFASs were as follows: 1.06 ± 0.78 ng/g for PFHxA, 1.02 ± 0.76 ng/g for PFHpA, 0.87 ± 0.68 ng/g for PFOA and below the method quantification limit for PFOS. Clear differences in PFASs levels were noted between the two regions.

**Conclusion:**

Sheep are exposed to various PFASs, and sheep wool and items made of it may be the source of human exposure to these compounds. Hair samples may be used for biomonitoring of sheep exposure to PFASs.

## Introduction

Poly- and perfluoroalkyl substances (PFASs) are a numerous group of synthetic organic compounds that contain at least one fully fluorinated methyl or methylene carbon atom (12). Their thermal and chemical stability make PFASs commonly chosen for applications in various industries, and they are contained in many everyday objects, such as clothes, domestic appliances, electronic equipment and many others (23). These compounds may permeate the natural environment, and existing research has shown PFASs to be in surface waters and groundwater, soil, dust and plants in various regions of the world (23, 25). It is known that PFASs may penetrate into human and animal organisms mainly through the digestive system, but also through the lungs and skin (23), and impact negatively when they do. Poly- and perfluoroalkyl substances have been found in blood serum, urine, milk, semen and various internal organs (13, 17). These substances chiefly disturb the activity of the hormonal system, and hence they are included among the compounds termed “endocrine-disrupting chemicals”. It is known that PFASs practically impair the functioning of the whole body, especially the reproductive, nervous, immunological and gastrointestinal systems (6, 23).

**Fig. 1.**
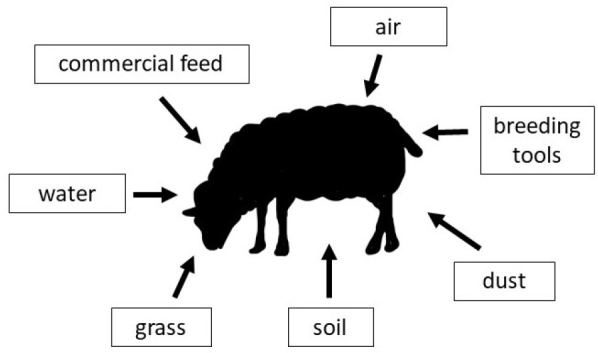
Sources of sheep’s exposure to poly- and perfluoroalkyl substances

The majority of previous data concern human exposure to PFASs, and knowledge of these compounds in farm animals is rather limited. Only two previous studies concern PFASs in sheep (16, 27). However, these works do not focus on PFAS biomonitoring in sheep but on the compounds’ distribution in the animal’s body after exposure. It is well known that PFASs pollute not only highly urbanised and industrialised regions, but also rural areas and regions, which have remained largely unaffected by human activity (24). This is because besides the compounds’ incorporation in manufactured products, they can arise in the atmosphere by photooxidation from various volatile precursor compounds which are common pollutants and may be transported in the air over long distances (9). In rural areas, PFASs may permeate the environment and be taken up into arable crops from residuals containing them (such as biosolids) used in agricultural plant protection products and irrigation water, the risk of which is significant given that such water is often reclaimed from wastewater (21, 26). These circumstances indicate that the sources of farm animal exposure to PFASs are various (Fig. 1). Such exposure not only deteriorates animal health, but also poses a potential threat to the humans who consume products of animal origin.

Among the matrices used to monitor PFASs in living organisms, hair samples are becoming increasingly important, helped by previous investigations which have proved that hair is an appropriate matrix for evaluation of the exposure to PFASs and that the levels in hair correlate with levels of these substances in internal organs (4, 8, 18). Hair is the best matrix to study long-term exposure to various environmental pollutants (including PFASs), because substances accumulate in the hair and their levels are not subject to rapid changes, as is the case in the classic matrices such as urine or serum (1, 4, 20). Moreover, hair sample collection is easy, completely non-invasive and stress-free.

The aim of this study was to assess sheep exposure to selected PFASs commonly used in industry (such as perfluorobutanoic acid – PFBuA, perfluoropentanoic acid – PFPeA, perfluorohexanoic acid – PFHxA, perfluoroheptanoic acid – PFHpA, perfluorooctanoic acid – PFOA and perfluorooctane sulfonic acid – PFOS) through hair sample analysis. It should be underlined that this is the first investigation concerning the evaluation of PFAS levels in sheep hair and the first study on PFASs in biological samples in the Kyrgyz Republic. The obtained results will contribute to expanding the knowledge of the exposure of farm animals to PFASs.

## Material and Methods

### Reagents

The reagents used during the study were the same as those used previously by Makowska *et al*. (18). The following reagents (all of analytical grade unless otherwise specified) were used: acetic acid (HAc), ammonium acetate and sodium dodecyl sulphate (SDS) (all obtained from Panreac, Barcelona, Spain); high-performance liquid chromatography–grade acetone, methanol and water (supplied by Romil, Barcelona, Spain); and PFBuA (98%), PFPeA (97%), PFHxA (≥97%), PFHpA (99%), PFOA (96%) and PFOS (≥98%) (all from Sigma-Aldrich, Steinheim, Germany). Perfluorooctanoic acid-13C4 (99%, used as an internal standard (IS)) was obtained from Cambridge Isotope Laboratories (Cambridge, MA, USA). Individual stock standard solutions were prepared at 1,000 mg/L in MeOH and stored at -18°C. Working solutions were prepared by dilution of the stock standard solutions in MeOH.

### Sample collection

The present study included 50 sheep (39 females and 11 males) of Kyrgyz and Arashan breeds ranging in age from 1 to 8 years (mean 2.1 ± 1.1). Hair samples were collected from animals bred in two regions of the Kyrgyz Republic: the Alamedin Region and the Sokuluk Region, both located in the vicinity of Bishkek – the capital and biggest city of the Kyrgyz Republic. The key characteristics of the regions are presented in Table 1. Twenty-five animals from each region were included in the experiment. Samples were collected in June of 2023. Approximately 2 g of hair was cut from the back of each animal above the shoulder blades as close to the skin as possible using metal scissors. Hair samples were placed in aluminium foil sachets directly after collection and kept in the dark at room temperature. Particular attention was paid to ensuring that the samples did not come into contact with objects that may contain PFASs.

**Table 1.** Key characteristics of the Kyrgyz Republic regions from which sheep hair was sampled for poly- and perfluoroalkyl substance determination

Region name	Alamedin	Sokuluk
Area (km^2^)	1,503	2,550
Human population in 2024 (in thousands)	180.6	250.6
Human population density (thousands/km^2^)	0.120	0.098
Administrative centre	Lebedinovka	Sokuluk
Distance in the straight line from the centre of Bishkek to administrative centre of the region (km)	9.50	22.29
Population concentrations	51 settlements in 17 rural communities	1 town and 68 settlements in 19 rural communities
Number of sheep and goats in 2015 ^*1*^, ^*2*^	123,019	51,033
Wool production in 2024 (in tonnes)	158	329.1

1 – data for sheep alone are not available;

2 – more recent data concern larger administrative areas: the Alamedin and Sokuluk regions are located in Chui Oblast, in which the number of sheep and goats amounted to 660,049 animals in 2022. The origin of the data is the National Statistical Committee of the Kyrgyz Republic (https://stat.gov.kg/ru/statistics/selskoe-hozyajstvo/)

Because hair sample collection is completely non-invasive, the Ethical Committee’s consent was not required to conduct this research. It is in accordance with the law in force in the Kyrgyz Republic and the European Union. The verbal consent of the animals’ owners was obtained for the use of their animals’ hair in the present study.

Before analysis, exogenous substances contaminating the hair surface were removed by the method previously described by Makowska *et al*. (18). Briefly, the hair was rinsed with ultrapure water, then in SDS (0.1%, w/v) and again twice in ultrapure water. Between each washing and the next, the samples were subjected to sonication for 5 min. After washing, the hair was cut into 2–3 mm fragments and stored for no longer than three days until further analysis.

### Hair sample analysis

The levels of PFASs in the sheep hair samples were evaluated according to the method previously described by Martín *et al*. (19) and Makowska *et al*. (18) after revalidation. A 100 mg mass of hair sample and 12.5 ng of the IS were placed in a glass centrifuge tube and incubated with 2 mL of a mixture of methanol and HAc (85 : 15, v/v) at 38°C for 12 h. Then the samples were allowed to cool to room temperature and 3 mL of acetone was added to them. The samples were sonicated for 15 min and centrifuged for 10 min at 2,900 × *g*. The organic phase containing the analytes was separated, transferred to a clean tube and evaporated to dryness under a nitrogen stream at room temperature. The residue was reconstituted with 0.25 mL of methanol and passed through a 0.22 μm nylon filter. A 10 μL aliquot of the extract was injected into the liquid chromatography–tandem mass spectrometry (LC-MS/MS) apparatus, which was a 1290 Infinity II ultra-high-performance liquid chromatography system (Agilent, Santa Clara, CA, USA).

Separation was carried out with a HALO C-18 Rapid Resolution column of 50 × 4.6 mm internal diameter with 2.7 μm particle size (Advanced Materials Technology, Wilmington, DE, USA) using a gradient elution with 10 mM ammonium acetate solution (solvent A) and methanol (solvent B) at a flow rate of 0.6 mL/min. The gradient was as follows: 0–14 min solvent B increased in linear fashion from 28 to 70%, 14–19 min its proportion rose from 70% to 80%, 19–25 min it increased to 100% and 25–27 min it was held. The column temperature was maintained at 30°C. Two multiple-reaction monitoring transitions were selected for each analysis for quantification and confirmation of compounds. The mass spectrometer settings and validation parameters are summarised in Table 2 and Table 3.

**Table 2.** Optimised tandem mass spectrometry parameters for multiple-reaction monitoring (MRM) analysis of Kyrgyz Republic sheep hair sampled for poly- and perfluoroalkyl substance determination

Compound	Precursor ion (*m/z*)	MRM 1^a^ (*m/z*)	MRM 2^b^ (*m/z*)	Fragmentor (V)	Collision energy (eV)
PFBuA	213	169.0	51.6	55	0
PFPeA	263	219.0	89.7	55	0
PFHxA	313	269.0	119.0	60	0
PFHpA	363	319.0	332.8	65	0
PFOA	413	369.0	194.3	62	0
PFOS	499	80.0	51.5	145	40
PFOA-^13^C_4_	417	371.9	172.0	77	4

a – transition used for quantification;

b – transition used for confirmation; PFBuA – perfluorobutanoic acid; PFPeA – perfluoropentanoic acid; PFHxA – perfluorohexanoic acid; PFHpA – perfluoroheptanoic acid; PFOA – perfluorooctanoic acid; PFOS – perfluorooctane sulphonic acid

**Table 3.** Linear range, method detection limits (MDLs), method quantification limits (MQLs), recovery and precision of the selected poly- and perfluoroalkyl substances analysed in hair samples of Kyrgyz Republic sheep

Compound	Linear range (ng/g)	MDL (ng/g)	MQL (ng/g)	Recovery (%)			Inter-day precision (RSD %)
				Low level	Medium level	High level	
PFBuA	0.9–100	0.3	0.9	96 ± 3	99 ± 3	100 ± 4	4
PFPeA	0.6–100	0.2	0.6	93 ± 5	93 ± 7	97 ± 5	3
PFHxA	0.6–100	0.2	0.6	88 ± 7	92 ± 8	92 ± 5	5
PFHpA	0.6–100	0.2	0.6	87 ± 6	86 ± 6	90 ± 6	3
PFOA	0.6–100	0.2	0.6	91 ± 2	90 ± 2	95 ± 2	4
PFOS	0.6–100	0.2	0.6	83 ± 7	88 ± 5	89 ± 4	5

1 RSD – relative standard deviation; PFBuA – perfluorobutanoic acid; PFPeA – perfluoropentanoic acid; PFHxA – perfluorohexanoic acid; PFHpA – perfluoroheptanoic acid; PFOA – perfluorooctanoic acid; PFOS – perfluorooctane sulphonic acid

### Quality assurance and quality control

A quality assurance and quality control protocol was designed to ensure reliable and precise results. It involved the use of spiked control samples, solvent (methanol) injections, standards containing a mixture of the target compounds (20 ng/mL) and procedural blanks (processed in the same way as the samples) in each analytical batch (which was of 15 samples, to preserve the stability of the chromatographic column). Each sample was injected in duplicate. No quantifiable amounts of target compounds were detected in the blank samples.

### Statistical analysis

GraphPad Prism version 9.2.0 (GraphPad Software, San Diego, CA, USA) was used to carry out statistical analysis. The statistical analysis included descriptive statistics and a non-parametric Mann–Whitney test (to compare PFAS levels between regions). The differences were considered statistically significant at P-value < 0.05. Concentrations of PFASs lower than the method quantification limit (MQL) were included in the statistics as MQL/2.

## Results

The compounds of interest were observed in all hair samples included in the study. The levels of two of them, *i.e*. PFPeA and PFBuA, were higher than the MQL in all samples. The concentration of PFPeA was the highest and ranged from 0.99 ng/g to 27.90 ng/g with a mean (± standard deviation) of 5.55 ± 4.54 ng/g. The concentrations of PFBuA were slightly lower – from 0.95 ng/g to 14.18 ng/g with a mean of 2.24 ± 2.34 ng/g. Levels above the MQL of PFHxA were noted in 72% of samples, and the highest concentration level of this substance found amounted to 3.87 ng/g (mean 1.06 ± 0.98 ng/g). Concentrations of PFHpA above the MQL were observed in fewer samples than such concentrations of PFHxA were (66%), but its maximum concentration was slightly higher (4.32 ng/g). The mean level of PFHpA was 1.02 ± 0.76 ng/g. The lowest concentrations found were of PFOA and PFOS. Levels of the former above the MQL were observed in 54% of samples, the highest level of this substance amounted to 2.67 ng/g and the mean value was 0.87 ± 0.68 ng/g. Levels of PFOS higher than the MQL were only discovered in two samples (4%) and were 1.92 ng/g and 3.36 ng/g. A summary of the results obtained is presented in Table 4.

**Table 4.** Concentration levels (ng/g), frequency of detection (%), method detection limit (MDL (ng/g)) and method quantification limit (MQL (ng/g)) of poly- and perfluoroalkyl substances (n = 50) – cumulative data from hair samples of Kyrgyz Republic sheep

Compound	Range	Arithmetic mean	Geometric mean	25th percentile	Median	75th percentile	Samples >MDL (%)	MDL	Samples >MQL (%)	MQL
PFBuA	0.95–14.18	2.24 ± 2.34	1.79	1.25	1.54	2.30	100	0.30	100	0.90
PFPeA	0.99–27.90	5.55 ± 4.54	4.50	3.33	4.18	5.85	100	0.20	100	0.60
PFHxA	< MQL–3.87	1.06 ± 0.78	0.82	<MQL	0.88	1.44	100	0.20	70	0.60
PFHpA	<MQL–4.32	1.02 ± 0.76	0.78	<MQL	<MQL	0.95	100	0.20	64	0.60
PFOA	<MQL–2.67	0.87 ± 0.68	0.65	<MQL	0.80	1.21	100	0.20	52	0.60
PFOS	<MQL–3.36	<MQL	<MQL	<MQL	<MQL	<MQL	100	0.20	4	0.60

1 PFBuA – perfluorobutanoic acid; PFPeA – perfluoropentanoic acid; PFHxA – perfluorohexanoic acid; PFHpA – perfluoroheptanoic acid; PFOA – perfluorooctanoic acid; PFOS – perfluorooctanesulfonic acid

**Fig. 2.**
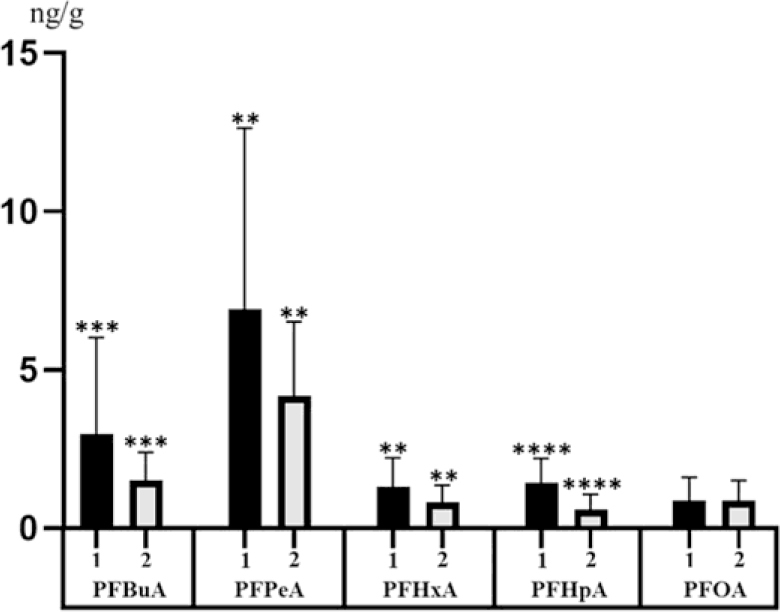
Mean concentration levels (± standard deviation) of perfluorobutanoic acid (PFBuA), perfluoropentanoic acid (PFPeA), perfluorohexanoic acid (PFHxA), perfluoroheptanoic acid (PFHpA) and perfluorooctanoic acid (PFOA) in sheep hair samples from the Kyrgyz Republic Alamedin (1) and Sokuluk (2) regions. * – P-value < 0.05; *** – P-value < 0.001; **** – P-value < 0.0001

Clear differences in PFAS levels in sheep hair were noted between districts included in the investigation. Generally higher levels of PFASs were noted in the Alamedin Region (Fig. 2).

The most visible differences between regions were associated with the levels of PFHpA and PFBuA. In the Alamedin Region the mean concentration level (±standard deviation) amounted to 1.45 ± 0.77 ng/g for PFHpA and 2.97 ± 3.05 ng/g for PFBuA, and in Sokuluk Region these values were 0.59 ± 0.47 ng/g and 1.51 ± 0.89 ng/g, respectively (Fig. 2). The observed differences were highly statistically significant (P-value < 0.0001 for PFHpA and P-value = 0.0001 for PFBuA). Less clear differences between regions emerged for PFPeA and PFHxA (Fig. 2). In the Alamedin Region, the mean concentration level of PFPeA was 6.92 ± 5.72 ng/g and the level of PFHxA was 1.30 ± 0.91 ng/g. In the Sokuluk Region, these values were found to be 4.19 ± 2.33 ng/g and 0.82 ± 0.43 ng/g, respectively. In this case, the differences were statistically significant (P-value = 0.0264 for PFPeA and P-value = 0.0421 for PFHxA). The mean concentration level of PFOA was 0.88 ± 0.73 ng/g in the Alamedin Region and 0.87 ± 0.63 ng/g in the Sokuluk Region, and this difference was not statistically significant (P-value = 0.9032) (Fig. 2). The number of samples with PFOS levels higher than the MQL was too small for comparison of the regions to be possible.

## Discussion

This study is the first report concerning PFAS levels in the hair of farm animals. Until now, the majority of previous studies on PFAS levels in the hair have been conducted on humans, and only single works describe these determinations in other mammal species (Table 5).

**Table 5.** The poly- and perfluoroalkyl substances observed in selected previous hair sample studies and included in this Kyrgyz Republic sheep hair sample study

Substance	n	Range (pg/mg)	Median/mean (pg/mg)^1^	Frequency detection^2^	Reference
Human hair					
Belgium					
PFPeA	114	<MQL	<MQL/-	6.1	(4)
PFHxA		<MQL–2.4	<MQL/-	18.4	
PFHpA		<MQL–1.4	<MQL/-	4.4	
PFOA		<MQL–3.4	<MQL/-	86.8	
PFOS		<MQL–11.3	<MQL/-	0.9	
China					
PFBuA	28	ND	-	0	(7)
PFPeA		ND–326	-/31.7	40	
PFHxA		ND–201	-/29.2	73	
PFHpA		ND–68.5	-/12.6	33	
PFOA		18.3–1390	-/277	100	
PFOS		1.01–51.0	-/6.76	100	
South Korea					
PFPeA	94	ND–0.75	<MDL/0.48	16	(14)
PFHxA		ND–0.14	<MDL/0.13	2	
PFHpA		ND–0.28	<MDL/0.14	26	
PFOA		1.3–9.8	3.2/3.6	100	
PFOS		0.69–15	4.2/4.8	100	
Spain					
PFBuA	42	5.8–15.5	7.8/8.9	14	(20)
PFPeA		4.6–13.3	6.8/7.4	26	
PFHxA		2.0–10.6	8.9/8.1	26	
PFHpA		0.6–10.1	2.4/3.1	86	
PFOA		0.6–9.5	1.4/2.0	57	
PFOS		0.7–11.0	1.9/2.7	76	
Dog hair					
Poland					
PFBuA	30	0.98–26.6	9.66/11.2	100	(18)
PFPeA		<0.14–15.6	4.39/5.31	96.7	
PFHxA		<0.14–18.9	5.92/7.45	96.7	
PFHpA		<0.14–13.2	1.35/2.10	86.7	
PFOA		1.51–66.7	17.4/21.2	100	
PFOS		<0.14–1.63	0.70/0.76	86.7	
Sheep hair					
Kyrgyz Republic					
PFBuA	50	0.95–14.8	1.54/2.24	100/100^2^	This study
PFPeA		0.99–27.90	4.18/5.55	100/100^2^	
PFHxA		<MQL–3.87	0.88/1.06	100/70^2^	
PFHpA		<MQL–4.32	<MQL/0.87	100/64^2^	
PFOA		<MQL–2.67	0.80/0.87	100/52^2^	
PFOS		<MQL–3.36	<MQL/<MQL	100/4^2^	

1 – research contains no values;

2 – % samples >MDL/% samples >MQL; <MDL – below method detection limit; <MQL – below method quantification limit; ND – not detected

Relatively few works have described PFASs in hair (Table 5), which is counter to expectations because this matrix seems to be very apt for such studies, especially for investigations on long-term exposure (28). This is because substances accumulate in the hair and do not undergo changes as quickly as in blood or urine. Moreover, it has been proved that hair is a suitable matrix in which to investigate PFAS levels (Table 5). A study on experimental animals has also shown that PFAS levels in hair reflect the extent of the animal’s exposure and depend on PFAS doses in sub-chronic exposure. Moreover, there are good positive correlations between PFAS levels in the hair and in serum and other tissues (8). Other advantages of hair samples are their non-invasive and stress-free collection and easy storage (5). Of course, analysis of hair has also some inconveniences. The most important of them is the possible penetration of substances into the structure of the hair not only internally though capillary vessels and the hair root, but also externally directly from the environment; during analysis it is impossible to separate these two penetration routes (28). Moreover, the hair sample analysis is not useful in studies on short-term changes in exposure to PFASs and other substances (28). However, despite these limitations, in the light of previous studies, hair analyses reflect the internal body burden of PFASs well and can be successfully used to evaluate human and animal exposure to these substances (Table 5).

The results obtained during the present study have shown that sheep are commonly exposed to various PFASs, which is similar to the situation observed in humans and dogs (Table 5). However, a precise comparison of PFAS levels observed in the present study with results obtained in previous investigations is difficult. This is because earlier research was performed in other parts of the world, and differences between studies have proved relatively well that the exposure of living organisms to PFASs depends on the regional environmental factors in play, including chiefly the degree of industrialisation and urbanisation (24), but also closer, local unidentified factors (10). The existence of local factors influencing exposure to PFASs is also confirmed by the present study, which has shown clear differences in the levels of PFASs in sheep hair between the two neighbouring regions where the research was carried out.

It would seem that levels of PFASs, as androgenic pollutants, would be dramatically higher in humans. Indeed some previous studies confirm this thesis. Nevertheless, the levels of PFASs found in the sheep during the present research are higher than those noted in human hair in some parts of the world (Table 5). It can be assumed that this correlates with the level of environmental pollution with PFASs in the regions where studies have been performed. Besides the levels, the profile of PFASs observed in sheep in this research is also different to that found in studies on human hair. Generally, in studies on humans PFOS and PFOA dominate, while in this research PFBuA and PFPeA were measured at the highest concentrations (Table 5). These differences may result from the multifactorial character of human exposure to PFASs, such as higher exposure during particular kinds of professional activity and more extensive exposure with certain lifestyle, clothing, personal care product and domestic fixture and fitting choices, none of which are relevant to animals (11). On the other hand, previous studies showed that PFOS was the dominant PFAS in the sheep liver (27), which in turn suggests that differences in the profiles of PFASs between the present and previous studies result from local factors influencing the natural environment’s pollution with PFASs in various regions of the world.

The comparison of the data obtained during this work with that of earlier studies concerning sheep exposure to PFASs is even more difficult. Until now there have only been two studies on this issue, conducted in other parts of the world and on other matrices (16, 27). In one of them, conducted on liver samples collected in the Netherlands, the presence of PFOS at levels from <0.5–4.5 ng/g was found, and levels of other PFASs (PFHxA, PFHpA and PFOA) were below the MQL (0.5 ng/g) (27). The second study primarily investigated how oral administration of PFOA and PFOS formed the levels of these substances in various parts of the sheep’s body (16). The control group in this experiment was only one animal, and the concentration levels of PFOS in this sheep were as follows: 0.21 μg/L in plasma, 1.53 μg/g in liver, 0.31 μg/g in kidney and below the method detection limit (MDL) in muscles. The levels of PFOA were below the MDL in all of these tissues (16). Such limited previous research in sheep makes it virtually impossible to compare the current results meaningfully with extant data. However, both the previous studies (16, 27) and the present investigations clearly indicate that sheep are exposed to PFASs. Moreover, previous observations have shown that the frequency of PFOS detection in sheep’s livers (above 77.8%) was much higher than that noted in cows’ (41%) or pigs’ livers (5%) (27), which suggests that sheep exposure to PFASs is considerable. This view was also supported by this study, which has shown the presence of all PFASs studied in 100% of the samples. The reason for such high exposure of sheep to PFASs is unknown, but it may result from the specificity of sheep breeding and the longer stay of these animals on pastures compared to other livestock’s pasturing.

The exposure of sheep and other farm animals to PFASs is associated with some risk to humans, who are consumers of products of animal origin. Previous studies on sheep have shown that even relatively short feeding (21 days) with food contaminated with PFASs increases the levels of these substances in blood plasma, milk, muscles and internal organs (16). Because the metabolism of PFASs in sheep is not fully understood, it is not known how the PFAS concentrations observed in the hair in this investigation correlate with the levels of these substances in muscles, internal organs or milk. In rats, however, observations on experimental animals have indicated that PFAS levels in hair demonstrate strong positive correlation with concentrations of these substances in internal organs (8). Therefore, it can be assumed that a similar situation occurs in sheep, and in animals in which PFASs are found in the hair, they are also present in body fluids and tissues. The presence of PFASs in milk or meat can be dangerous for people. Also, the present study has clearly proved that sheep wool and items made of it may be a source of exposure to PFASs in humans. This is all the more important as PFASs may penetrate into the organism not only through the digestive tract or lungs, but also by absorption through the skin (3).

The question also arises of whether the levels of PFASs noted in the present study can have any consequences for animal health. It should be pointed out that no correlations between PFAS levels in the hair and their impact on the organism are yet known even in humans. There is also precious little elucidated in the animal kingdom: the metabolism of PFASs and their distribution in the sheep organism are known to an extremely limited extent (13, 27). For these reasons the impact of the observed PFAS levels on animals cannot be clearly determined. Nevertheless, the strong possibility of such an impact cannot be excluded because it is known that even in low doses, PFASs adversely affect various organs and systems (2, 15).

## Conclusion

In this research hair samples have been used to evaluate sheep exposure to PFASs. The obtained results have indicated that sheep in the Kyrgyz Republic are exposed to various PFASs. All PFASs included in the study were found in the sheep hair samples; PFPeA and PFBuA were found in heaviest presence. Clear differences between PFAS levels in hair samples collected from one region and the levels in the samples from the other were noted. The present study has also shown that sheep wool and items made of it may be a source of PFASs in humans. Keeping in mind the positive correlation of previous studies’ PFAS levels in hair with levels of these substances in internal organs, this research has also shown that hair analysis may be utilised not only in the assessment of exposure of sheep to PFASs, but also in the estimation of the risk of human exposure to PFASs connected with the consumption of products of animal origin. However, many aspects of sheep’s exposure to PFASs are not clear and their explanation requires further comprehensive research.
